# The blood pressure-elevating effect of Red Bull energy drink is mimicked by caffeine but through different hemodynamic pathways

**DOI:** 10.14814/phy2.12290

**Published:** 2015-02-25

**Authors:** Jennifer L Miles-Chan, Nathalie Charrière, Erik K Grasser, Jean-Pierre Montani, Abdul G Dulloo

**Affiliations:** Laboratory of Integrative Cardiovascular and Metabolic Physiology, Division of Physiology, Department of Medicine, University of FribourgFribourg, Switzerland

**Keywords:** Blood pressure, caffeine, energy drink, hemodynamics

## Abstract

The energy drink Red Bull (RB) has recently been shown to elevate resting blood pressure (BP) and double product (reflecting increased myocardial load). However, the extent to which these effects can be explained by the drink's caffeine and sugar content remains to be determined. We compared the cardiovascular impact of RB to those of a comparable amount of caffeine, and its sugar-free version in eight young healthy men. Participants attended four experimental sessions on separate days according to a placebo-controlled randomized crossover study design. Beat-to-beat hemodynamic measurements were made continuously for 30 min at baseline and for 2 h following ingestion of 355 mL of either (1) RB + placebo; (2) sugar-free RB + placebo; (3) water + 120 mg caffeine, or (4) water + placebo. RB, sugar-free RB, and water + caffeine increased BP equally (3–4 mmHg) in comparison to water + placebo (*P* < 0.001). RB increased heart rate, stroke volume, cardiac output, double product, and cardiac contractility, but decreased total peripheral resistance (TPR) (all *P* < 0.01), with no such changes observed following the other interventions. Conversely, sugar-free RB and water + caffeine both increased TPR in comparison to the water + placebo control (*P* < 0.05). While the impact of RB on BP is the same as that of a comparable quantity of caffeine, the increase occurs through different hemodynamic pathways with RB's effects primarily on cardiac parameters, while caffeine elicits primarily vascular effects. Additionally, the auxiliary components of RB (taurine, glucuronolactone, and B-group vitamins) do not appear to influence these pathways.

## Introduction

Despite the increasing popularity and consumption of energy drinks worldwide (Heckman et al. 2010), there has been surprisingly little robust investigation into their effects on the cardiovascular system. The research which has been undertaken is often focused on sport or cognitive performance (for reviews, see Mora-Rodriguez and Pallares 2014; Childs 2014), lacks tight control of food and beverage consumption prior to testing (Franks et al. 2012), and the hemodynamic measurements are infrequent during the postdrink period making the detection of small-to-modest changes problematic (Alford et al. 2001; Baum and Weiss 2001; Ragsdale et al. 2010; Menci et al. 2013).

Using continuous, beat-to-beat cardiovascular monitoring, we recently reported that, in young healthy subjects, the energy drink Red Bull (RB) elevates blood pressure (BP) and double product (DP) at rest – the latter reflecting increased myocardial load at rest (Grasser et al. 2014b), and in response to a mental stress task (Grasser et al. 2015). Such observed increases in response to a commercially- and readily available drink may have clinical importance in individuals with existing heart conditions, or in chronic energy drink consumers. However, the extent to which these effects can be explained by the drink's caffeine content remains to be determined. Therefore, we compared the cardiovascular impact of RB energy drink to those of a comparable amount of caffeine, which was ingested in the form of a capsule or as sugar-free RB (and thus in combination with the other drink components), with the continuous beat-to-beat measurement of hemodynamic profile.

## Subjects and Methods

Eight young, healthy men of European descent were recruited from the local student population and participated in the present study, with a mean (±SE) age of 25.4 ± 1.3 years, weight of 75.6 ± 3.9 kg, and body mass index (BMI) of 24.4 ± 1.0 kg/m^2^. All subjects were weight-stable, with less than 3% body weight variation in 6 months preceding the study. Smokers, claustrophobic individuals, individuals taking medication, those with any metabolic disease, and caffeine naïve individuals were excluded. Daily caffeine intake (estimated by questionnaire) ranged between 100 and 350 mg/day (mean = 210 ± 30 mg/day). Only one of the eight subjects consumed energy drinks on a regular basis (i.e., >1 per week). Each subject completed four separate experimental test days, according to a randomized crossover design, with at least a 2-day interval between any two test days. The concomitant metabolic response was measured in all subjects on each of the four test days, with the metabolic data for three of the four test substances previously published specifically in the context of the effect of sugar-free RB, and its potentially bioactive ingredients, on thermogenesis (Miles-Chan et al. 2015). The study complied with the Helsinki Declaration of 1975 as revised in 1983 and was approved by the state ethical review board; all participants gave written consent.

### Experimental design

Prior to testing, participants visited the laboratory in order to complete a questionnaire regarding their lifestyle and medical history, and to familiarize themselves with the experimental procedure and equipment. All participants were requested to avoid physical activity, caffeine, and dietary supplements in 24 h prior to testing. Furthermore, in order to minimize the effect of physical activity on the morning of each test day, participants were requested to use motorized transport instead of walking or cycling to reach the laboratory. On the day of testing, participants arrived at the laboratory at 8.00 following a 12 h overnight fast. After the participant voided their bladder, body weight and height were measured using a mechanical column scale with integrated stadiometer (Seca model 709, Hamburg, Germany). Participants were seated comfortably in a car seat adapted for cardiovascular monitoring, and the monitoring equipment was connected. A baseline measurement was conducted as described below until stability of the cardiovascular parameters for at least 30 min. During this period, the participant was instructed to relax and avoid movements. The subject then ingested one of the following four test substances, at a convenient pace over 4 min:

1. 355 mL of degassed commercially available energy drink (RB) + placebo capsule;
2. 355 mL of a degassed, sugar-free version of the RB energy drink + placebo capsule (sfRB);
3. 355 mL of distilled water + capsule containing an equivalent amount (120 mg) of caffeine (W + caff);
4. 355 mL of distilled water + placebo capsule (W + P);

It should be noted that although the quantity of caffeine within the capsules (120 mg) was slightly higher than that stated by RB as being present within its beverages (114 mg per 355 mL), it is the average value of caffeine content reported through independent analyses performed in recent years, which range from 115 to 124 mg per 355 mL serving (Nour et al. 2010; Hassan and Al-Abbad 2011; Ali et al. 2012; Jenway Analysis 2012; Vochyanova et al. 2014; Sereshti and Samadi 2014). For a detailed list, and comparison, of the nutrient composition of RB and sfRB, please refer to Table1.

**Table 1:** Composition details of Red Bull (RB) and Sugar-free Red Bull (sfRB)

	Unit	Manufacturer per 100 mL		USDA (2014) per 100 g	
		RB	sfRB	RB	sfRB
Proximates					
Water	g			88.45	98.35
Energy	kcal	45	3	45	5
Energy	kJ	192	14	190	19
Protein	g	0	0	0.25	0.25
Total lipid (fat)	g	0	0	0.08	0.08
Ash	g			0.28	0.28
Carbohydrate	g	11	0	10.94	0.7
Sugars, total	g			10.06	0
Minerals					
Calcium, Ca	mg			13	13
Iron, Fe	mg			0.02	0.02
Magnesium, Mg	mg			3	3
Potassium, K	mg			3	3
Sodium, Na	mg	40	40	83	83
Copper, Cu	mg			0.005	0.005
Manganese, Mn	mg			0.003	0.003
Selenium, Se	*μ*g			0.2	0.2
Vitamins					
Thiamin	mg			0.025	0.025
Riboflavin	mg			0.575	0.575
Niacin	mg	8	8	8.5	8.5
Panthothenic acid	mg	2	2	1.959	1.9
Vitamin B-6	mg	2	2	1.959	1.995
Choline, total	mg			0.3	0
Vitamin B-12	*μ*g	2	2	1.96	1.99
Other					
Caffeine	mg	32	32	31	31

*Ingredients of Red Bull (RB) energy drink (according to manufacturer):* Water, sucrose, glucose, acidity regulator (sodium citrate, magnesium carbonate), carbonic acid, acidifying agent: citric acid, taurine (400 mg/100 mL), caffeine (32 mg/100 mL), glucuronolactone (24 mg/100 mL), inositol, vitamins (niacin, panthothenic acid, B6, B12), flavor, color (caramel, riboflavin).

*Ingredients of Sugar-free Red Bull (sfRB) energy drink (according to manufacturer):* Water, acidity regulator (sodium citrate, magnesium carbonate), carbonic acid, acidifying agent: citric acid, taurine (400 mg/100 mL), caffeine (32 mg/100 mL), glucuronolactone (24 mg/100 mL), inositol, vitamins, flavor, color (caramel, riboflavin), sweeteners (aspartame, acesulfame-K), thickener: xanthan.

The postdrink cardiovascular monitoring continued for a further 120 min. In order to reduce boredom and accompanying stress and prevent sleeping, participants were permitted to watch a calm movie or a documentary throughout the measurements. All participants were blinded as to the order in which they would receive the test substances.

### Hemodynamic measurements

Beat-to-beat cardiovascular recordings were performed using a *Task Force Monitor* (*CNSystems*), with data sampled at a rate of 1000 Hz (Girona et al. 2014). ECG/Impedance electrodes were positioned together with upper arm and finger BP cuffs. Electrode strips were placed at the neck and thoracic regions, the latter specifically midclavicular at the xiphoid process level (Standard electrode kit, *CNSystems*, Medizintechnik, Graz, Austria). BP was monitored continuously using the Penaz principle from either the index or the middle finger of the right hand and was calibrated to oscillometric brachial BP measurements on the contralateral arm. The right hand rested on a ductile pillow which was positioned at heart level on a height adjustable table. Impedance cardiography measurements, in which the changes in thoracic impedance are converted to reflect changes in thoracic fluid content/volume over time, were performed based on the original Kubicek approach (Kubicek et al. 1966, 1970) but using an improved estimate of thoracic volume (Fortin et al. 2006), which allows calculation of cardiac stroke volume (SV). Index of contractility (IC), a marker of myocardial contractility (Grasser et al. 2009), was derived through impedance cardiography and reflects the aortic peak flow.

Heart rate (HR) was calculated from the appropriate RR-Interval. Cardiac output (CO) was computed as the product of SV and HR. Mean arterial BP (MAP) was calculated from diastolic BP (DBP) and systolic BP (SBP) as follows: MAP = DBP + ⅓(SBP − DBP). Total peripheral resistance (TPR) was calculated as MAP/CO. Double (rate pressure) product (DP) was calculated as HR × SBP and provides valuable information concerning the oxygen consumption of the myocardium (van Vliet and Montani 1999).

### Statistical analysis

The number of required subjects (*n* = 8) was determined by power analysis using the web software (http://www.statisticalsolutions.net/pss_calc.php) and based on a physiologically relevant 5 mmHg change in MAP and a standard deviation of 5 mmHg of the population (values chosen from our previous studies). We chose a type I error (*α*) of 0.05 and a desired power (1 − *β*) of 0.80.

**Figure 1:**
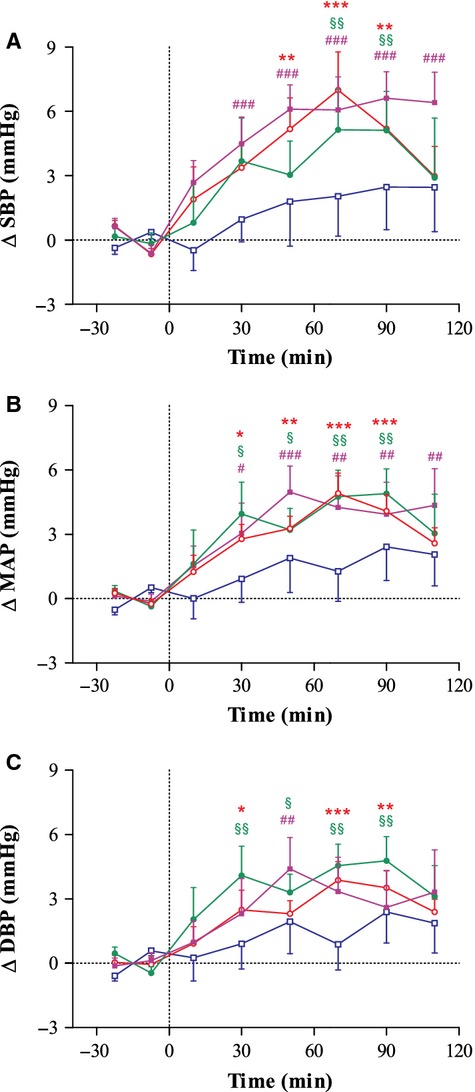
Time course of changes in systolic blood pressure (SBP; A), mean arterial blood pressure (MAP; B), and diastolic blood pressure (DBP; C) before and after ingestion of Red Bull + placebo (RB; ○), sugar-free Red Bull + placebo (sfRB; •), water + 120 mg caffeine (W + caff; ▪), or water + placebo (W + P; □). Mean ± SEM. Data analyzed using repeated measures ANOVA followed by Dunnett's multiple comparison test versus either baseline or W + P. Significantly different to baseline: RB: *P < 0.05, **P < 0.01, ***P < 0.001; sfRB: ^§^*P* < 0.05, ^§§^*P* < 0.01; W + caff: ^#^*P* < 0.05, ^##^*P* < 0.01, ^###^*P* < 0.001. *Overall ANOVA*: SBP = ANOVA, *P* < 0.001; Pairwise: all versus W + P, *P* < 0.001. MAP = ANOVA, *P* < 0.001; Pairwise: all versus W + P, *P* < 0.01. DBP = ANOVA, *P* < 0.001; Pairwise: all versus W + P, *P* < 0.01.

**Figure 2:**
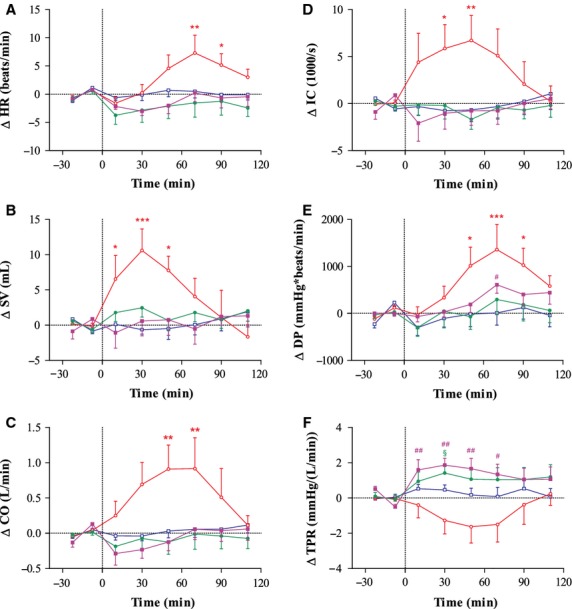
Time course of changes in heart rate (HR; A), stroke volume (SV; B), cardiac output (CO; C), index of contractility (IC; D), double product (DP; E), and total peripheral resistance (TPR; F) before and after ingestion of Red Bull + placebo (RB; ○), sugar-free Red Bull + placebo (sfRB; •), water + 120 mg caffeine (W + caff; ▪), or water + placebo (W + P; □). Mean ± SEM. Data analyzed using repeated measures ANOVA followed by Dunnett's multiple comparison test versus either baseline or W + P. Significantly different to baseline: RB: *P < 0.05, **P < 0.01, ***P < 0.001; sfRB: ^§^*P* < 0.05; W + caff: ^#^*P* < 0.05, ^##^*P* < 0.01. *Overall ANOVA:* HR, SV, CO, IC and DP = all ANOVAs, *P* < 0.001; Pairwise: RB versus W + P, *P* < 0.01; TPR = ANOVA, *P* < 0.001; Pairwise: RB versus W + P, *P* < 0.01, sfRB versus W + P, *P* < 0.05, W + caff versus W + P, *P* < 0.01.

Values of the cardiovascular recordings were averaged in 15 min epochs during the baseline and 20 min epochs for the 2-h postdrink period (in Figs. 1 and 2, graphical symbols have been placed at the midpoints of each of these epochs). All data are presented as mean ± SEM. The statistical treatment of data, by repeated measures ANOVA followed by Dunnett's multiple comparison test, was performed using the computer software Prism (Version 5.02, GraphPad Software Inc., San Diego, CA).

## Results

The baseline values of the cardiovascular recordings are presented in Table** **2, with no significant differences found in any of these predrink values between test days. No subject reported gastrointestinal symptoms or other unpleasant effects after ingestion of the drinks/capsules.

**Table 2:** Baseline hemodynamic data recorded prior to drink/capsule ingestion.

	RB		sfRB		W + caff		W + P	
	Mean	SEM	Mean	SEM	Mean	SEM	Mean	SEM
Systolic blood pressure (mmHg)	123	3	120	3	122	4	121	3
Mean blood pressure (mmHg)	93	2	90	2	94	3	93	2
Diastolic blood pressure (mmHg)	75	3	74	2	77	3	75	2
Heart rate (beats/min)	62	2	64	4	61	3	60	2
Double product (mmHg × beats/min)	7435	371	7660	569	7420	453	7555	544
Stroke volume (mL)	86	1	83	4	87	5	84	4
Cardiac output (L/min)	5.26	0.19	5.25	0.20	5.28	0.24	5.00	0.18
Total peripheral resistance (mmHg L^−1^ min^−1^)	17.3	0.8	16.9	0.8	17.3	0.6	18.0	0.7
Index of contractility (1000/sec)	53	3	51	4	53	4	50	3

RB, Red Bull + placebo; sfRB, sugar-free Red Bull + placebo; W + caff, water + 120 mg caffeine; W + P, water + placebo; *n *=* *8.

Changes in BP are presented in Figure 1. All the drink/caffeine combinations increased MAP in comparison to W + P (*P* < 0.001). These differences observed in both SBP (*P* < 0.01) and DBP (*P* < 0.01), beginning approximately 30 min after ingestion, with peak changes observed between 50 and 70 min. No significant change in BP from baseline was observed following ingestion of W + P.

Changes in HR, SV, CO, IC, DP, and TPR following ingestion of each drink/caffeine combination are shown in Figure 2. W + P did not have any significant effect on these parameters, with no significant differences from baseline observed during the 120-min postingestion period.

RB was the only treatment to increase HR, SV, CO, DP, IC (all *P* < 0.01), but also decreased TPR in comparison to the W + P control (*P* < 0.01). In contrast to RB, both sfRB (*P* < 0.05) and W + caff (*P* < 0.01) increased TPR in comparison to W + P.

There were no significant correlations between habitual caffeine consumption on any of the measured cardiovascular parameters (baseline values or postingestion response; see Table3), except for a statistically negative correlation (*r* = −0.76, *P* = 0.04) between habitual caffeine consumption and mean change in IC after RB ingestion.

**Table 3:** Spearman correlation statistics for estimated habitual caffeine consumption versus (A) baseline (B) post-ingestion mean response

	RB	sfRB	W+caff	W+P
(A)				
Baseline SBP	*r* = 0.05, *P* = 0.93	*r* = −0.19, *P* = 0.66	*r* = −0.14, *P* = 0.75	*r* = −0.10, *P* = 0.84
Baseline MAP	*r* = 0.17, *P* = 0.70	*r* = 0.14, *P* = 0.75	*r* = 0.31, *P* = 0.46	*r* = −0.02, *P* = 0.98
Baseline DBP	*r* = −0.21, *P* = 0.62	*r* = −0.19, *P* = 0.66	*r* = 0.02, *P* = 0.98	*r* = −0.10, *P* = 0.84
Baseline HR	*r* = 0.00, *P* = 1.02	*r* = −0.12, *P* = 0.79	*r* = 0.43, *P* = 0.30	*r* = 0.21, *P* = 0.62
Baseline SV	*r* = −0.14, *P* = 0.75	*r* = −0.10, *P* = 0.84	*r* = −0.69, *P* = 0.07	*r* = 0.07, *P* = 0.88
Baseline CO	*r* = −0.33, *P* = 0.43	*r* = −0.14, *P* = 0.75	*r* = −0.12, *P* = 0.79	*r* = −0.14, *P* = 0.75
Baseline IC	*r* = −0.21, *P* = 0.62	*r* = −0.24, *P* = 0.58	*r* = −0.31, *P* = 0.46	*r* = −0.21, *P* = 0.62
Baseline DP	*r* = −0.12, *P* = 0.79	*r* = −0.10, *P* = 0.84	*r* = 0.10, *P* = 0.84	*r* = 0.05, *P* = 0.93
Baseline TPR	*r* = 0.14, *P* = 0.75	*r* = 0.26, *P* = 0.54	*r* = 0.24, *P* = 0.58	*r* = 0.10, *P* = 0.84
(B)				
Δ SBP	*r* = −0.36, *P* = 0.39	*r* = 0.40, *P* = 0.33	*r* = −0.12, *P* = 0.79	*r* = −0.21, *P* = 0.62
Δ MAP	*r* = 0.02, *P* = 0.98	*r* = −0.36, *P* = 0.39	*r* = −0.20, *P* = 0.62	*r* = −0.26, *P* = 0.54
Δ DBP	*r* = 0.29, *P* = 0.50	*r* = −0.43, *P* = 0.30	*r* = −0.07, *P* = 0.88	*r* = −0.26, *P* = 0.54
Δ HR	*r* = −0.12, *P* = 0.79	*r* = −0.33, *P* = 0.43	*r* = −0.69, *P* = 0.07	*r* = 0.14, *P* = 0.75
Δ SV	*r* = −0.62, *P* = 0.11	*r* = −0.23, *P* = 0.50	*r* = 0.05, *P* = 0.93	*r* = −0.14, *P* = 0.75
Δ CO	*r* = −0.29, *P* = 0.50	*r* = −0.36, *P* = 0.39	*r* = −0.36, *P* = 0.39	*r* = −0.05, *P* = 0.93
Δ IC	*r* = −0.76, *P* = 0.04	*r* = 0.14, *P* = 0.75	*r* = −0.40, *P* = 0.33	*r* = −0.14, *P* = 0.75
Δ DP	*r* = −0.19, *P* = 0.66	*r* = −0.52, *P* = 0.20	*r* = −0.43, *P* = 0.30	*r* = −0.29, *P* = 0.50
Δ TPR	*r* = 0.12, *P* = 0.79	*r* = 0.31, *P* = 0.46	*r* = 0.55, *P* = 0.17	*r* = 0.21, *P* = 0.62

RB, RED BULL + placebo; sfRB, sugar-free RED BULL + placebo; W + caff, water + 120 mg caffeine; W + P, water + placebo; SBP, systolic blood pressure; MAP, mean arterial pressure; DBP, diastolic blood pressure; HR, heart rate; SV, stroke volume: CO, cardiac output; IC, index of contractility; DP, double product; TPR, total peripheral resistance. *n=*8.

## Discussion

To the best of our knowledge, this is the first study using beat-to-beat cardiovascular monitoring to compare the hemodynamic impact of RB energy drink to those of a comparable amount of caffeine, ingested in either capsule form or as a sfRB (and thus, in combination with the other drink components). Interestingly, while the impact of RB on BP appeared to be the same as that of a comparable quantity of caffeine, the increase affected by RB occurred through different hemodynamic pathways to that affected by W + caff or sfRB.

While the observed MAP elevations (3–4 mmHg), which peaked around 80 min postdrink, seem rather small, it has been found that even small sustained increases in BP elevate the vascular disease mortality risk among people who are considered as normotensives (Lewington et al. 2002), and are thus of relevance to chronic or binge energy drink consumers. However, whether acute changes are associated with cardiovascular mortality risk has still to be established.

The effects of RB appear to be largely on cardiac parameters; with increases in HR and SV (and hence CO), an elevation in DP which indicates increased workload, and an increase in IC indicating increased myocardial contractility. These observations are in agreement with those of our previous study (Grasser et al. 2014b) and of others (Steinke et al. 2009). Furthermore, as the ingredients, other than sugar and artificial sweeteners, appear to be very similar in both the regular and sugar-free versions of RB (see Table1), it is tempting to speculate that the differential hemodynamic effects of RB are predominately due to a synergistic response to sugar in combination with caffeine or any other RB components (taurine, glucuronolactone, and B-group vitamins), and are possibly mediated via insulin, a known positive cardiac inotrope (Klein and Visser 2010) which exerts its sympathoexcitatory effect via direct stimulation of the hypothalamus (Muntzel et al. 1994). This notion is supported by a recent study of Menci et al. (2013) who demonstrated a possible positive inotropic effect of RB (increases in both right and left ventricular myocardial function) in a similar population to that used in the present study (young, healthy subjects), and who consumed a similar volume of RB (approximately 300 mL). However, Menci et al. suggested this effect may have been due to taurine. Although the present study was not designed to test this hypothesis, our findings showing very similar hemodynamic pathways being influenced by sfRB and W + caff, suggest it is unlikely that taurine was responsible for, or enhanced, the pressor effect of RB. On the contrary, taurine has been shown to decrease both systolic and diastolic blood pressure (Fujita et al. 1987), and, at a population level, dietary taurine is associated with decreased risk of CVD (Yamori et al. 2010); thus leading to the European Union's Scientific Committee on Food to summarize that, in relation to the cardiovascular effects of energy drinks, “if there are any interactions between caffeine and taurine, taurine might reduce the cardiovascular effects of caffeine” (Scientific Committee on Food; European Commission 2003) (for a recent review of evidence, see Schaffer et al. 2014).

While a sugar-only test was not included in the present study, recent work in this laboratory (Grasser et al. 2014a) has shown no change in BP following ingestion of 60 g of sucrose (1.5× the sugar content contained within the volume of RB investigated here), thereby suggesting that the acute BP-elevating effect of RB is likely to be due to an interaction between sugar and caffeine on the hemodynamic system rather than sugar per se. Although plasma insulin and glucose were not measured in the present study (in order to avoid subtle hemodynamic changes pertaining to venous cannulation; Langham and Harrison 1993), and the sugar content of RB was relatively modest (∼40 g combined glucose and sucrose), it is well recognized that caffeine reduces whole body glucose disposal in a dose-dependent manner, with every mg/kg of body weight of caffeine ingested causing a 5.8% increase in the insulin area under the curve (Beaudoin et al. 2013). Thus, the average dosage of caffeine consumed here (1.6 ± 0.1 mg/kg) could well have been sufficient to elicit the observed hemodynamic response. Furthermore, despite a lack of information regarding the blood glucose profile in response to RB consumption, and its sugar-free equivalent, in healthy individuals, a recent study in type 1 diabetics (Olateju et al. 2015) shows a sustained, RB-induced (750 mL) increase in blood glucose, with peak blood glucose concentrations coinciding with the maximal pressor response observed in the present study (60–90 min). The response to 750 mL of a caffeine-free beverage matched for carbohydrate content was much lower, with no change in blood glucose following consumption of 750 mL of sfRB (Olateju et al. 2015). However, whether this holds true for a young, healthy population remains to be investigated.

In contrast to RB, both sfRB and W + caff increased TPR in comparison to W + P, with no evident change in CO, indicating that the elevations in BP in response to caffeine (in the absence of sugar) occurred largely through vascular mechanisms. This finding is supported by numerous other studies, such as those of Pincomb et al. (1985), and Sung et al. (1990)), who observed a decrease in HR and increase in BP and peripheral vascular resistance, with no change in stroke volume, cardiac output, or contractility, in response to 3.3 mg/kg of caffeine (a dose of approximately 220 mg) in healthy young men. In addition, more recent, specific studies of ventricular function have found no evidence for a positive inotropic effect of caffeine on myocardial cells (Leite-Moreira et al. 1999; Giacomin et al. 2008). As the effects of sfRB were largely the same as those of W + caff, it is likely that these effects were due to caffeine alone, with little or no influence of the auxiliary components (taurine, glucuronolactone and B-group vitamins).

Not all studies evaluating the cardiovascular impact of energy drinks have observed increases in BP parameters. For example, Ragsdale et al. (2010) reported no changes in BP (assessed by sphygmomanometry at 0, 60, 120 min postdrink) in response to ingestion of 250 mL of RB. However, a more in-depth analysis of their findings reveals a nonsignificant 3 mmHg increase in pressure 60-min postingestion, with no increase in response to the control drink. Therefore, one cannot disregard the possibility that such a tendency may have been detected as a significant increase if measured by continuous BP monitoring over a 2-h test period as was used in the present study. Moreover, our study was conducted in young, healthy males with moderate habitual caffeine consumption, and it therefore remains to be investigated whether such findings are applicable to other population and/or patient groups. Indeed, in light of concerns regarding the safety of energy drinks in vulnerable populations and chronic consumers (Higgins et al. 2010; Breda et al. 2014; Goldfarb et al. 2014), the observed increases in BP following RB ingestion may be of clinical relevance.

In conclusion, our results show that while the impact of RB on BP is the same as that of a comparable quantity of caffeine, the increase occurs through different hemodynamic pathways – with RB's effects apparently myocardial, while caffeine elicited vascular effects. As demonstrated by the similarity between the influence of sfRB and W + caff, the other components of RB (e.g., taurine) do not appear to influence these pathways. Therefore, we suggest that the differential hemodynamic origin of the pressor effect of RB versus caffeine is most likely due to an interaction of sugar with caffeine or any other RB components and warrants further investigation.
